# Genomics, microRNA, epigenetics, and proteomics for future diagnosis, treatment and monitoring response in upper GI cancers

**DOI:** 10.1186/s40169-016-0093-6

**Published:** 2016-04-06

**Authors:** Björn L. D. M. Brücher, Yan Li, Philipp Schnabel, Martin Daumer, Timothy J. Wallace, Rainer Kube, Bruno Zilberstein, Scott Steele, Jan L. A. Voskuil, Ijaz S. Jamall

**Affiliations:** Theodor-Billroth-Academy®, Munich, Germany; Theodor-Billroth-Academy®, Sacramento, CA USA; INCORE, International Consortium of Research Excellence of the Theodor-Billroth-Academy®, Munich, Germany; INCORE, International Consortium of Research Excellence of the Theodor-Billroth-Academy®, Sacramento, CA USA; Bon Secours Cancer Institute, Richmond, VA USA; Department of Surgery, Carl-Thiem-Klinikum, Cottbus, Germany; Proteogenomics Research Institute for Systems Medicine, San Diego, CA USA; Institute of Pathology, University of Homburg Saar, Homburg, Germany; Sylvia Lawry Center for MS Research, Munich, Germany; Department of Surgery, University of Sao Paulo, São Paulo, Brazil; Case Western Reserve University, Cleveland, OH USA; Department of Surgery, Madigan Army Medical Center, Tacoma, WA USA; Everest Biotech Ltd., Upper Heyford, Oxford, UK; Risk-Based Decisions, Inc., Sacramento, CA USA

**Keywords:** Genomics, MicroRNA, Epigenetics, Proteomics, Multimodal therapy, Response, Esophageal carcinoma, Esophageal squamous cell carcinoma, Esophageal adenocarcinoma, Barrett carcinoma, Upper gastrointestinal tract, Neoadjuvant therapy, Prognosis, Survival

## Abstract

One major objective for our evolving understanding in the treatment of cancers will be to address how a combination of diagnosis and treatment strategies can be used to integrate patient and tumor variables with an outcome-oriented approach. Such an approach, in a multimodal therapy setting, could identify those patients (1) who should undergo a defined treatment (personalized therapy) (2) in whom modifications of the multimodal therapy due to observed responses might lead to an improvement of the response and/or prognosis (individualized therapy), (3) who might not benefit from a particular toxic treatment regimen, and (4) who could be identified early on and thereby be spared the morbidity associated with such treatments. These strategies could lead in the direction of precision medicine and there is hope of integrating translational molecular data to improve cancer classifications. In order to achieve these goals, it is necessary to understand the key issues in different aspects of biotechnology to anticipate future directions of personalized and individualized diagnosis and multimodal treatment strategies. Providing an overview of translational data in cancers proved to be a challenge as different methods and techniques used to obtain molecular data are used and studies are based on different tumor entities with different tumor biology and prognoses as well as vastly different therapeutic approaches. The pros and cons of the available methodologies and the potential response data in genomics, microRNA, epigenetics and proteomics with a focus on upper gastrointestinal cancers are considered herein to allow for an understanding of where these technologies stand with respect to cancer diagnosis, prognosis and treatment.

## Introduction

According to the surveillance, epidemiology and end results (SEER) database, the estimated incidence for the USA of male-predominant upper gastrointestinal (GI) cancer (esophagus and gastric) includes 13.6 % of cancers of the digestive system and 2.4 % of all cancer sites with an observed peak in cancer rates in 1991 followed by a decrease of 24 % in men and 16 % in women through 2009, but esophageal cancer remains the 5th leading cause of cancer deaths in males between the ages of 40 and 59 years [1]. Elsewhere, Iranian colleagues reported the high incidence of esophageal cancer as an *esophageal cancer belt* comparing it with the incidence of laryngeal cancer [2]. The high incidences of upper GI cancers in northern Iran, Kazakhstan, northern central China, (especially Linxian Province), Japan and Singapore [3] can be thought of as an *upper GI cancer terrestrial belt*.

Despite many variables [4] epidemiologic observations are of value. Since the identification of *Helicobacter pylori (H. pylori)*, the incidences in gastric cancers have decreased worldwide, but there are reports of increases in esophageal squamous cell carcinomas (ESCC) as well as of adenocarcinomas of the esophago-gastral junction [5]. On the other hand, there has been a shift of distal gastric carcinomas to the proximal area of subcardial adenocarcinoma of the esophago-gastral junction (AEG Type III) and cardia localization (AEG Type II) over an 80-year period. This shift in tumor localization and its differences with rising incidences in the US or Asia are not well understood [5, 6] and provides an epidemiological challenge to identify and explain the worldwide differences in order to create effective preventive strategies. Eradication therapy for *H. pylori* and/or Barrett’s metaplasia alone cannot be the sole explanation as metaplasia of the esophago-gastric mucosa results in just 2 % mortality within 10 years of diagnosis [7]. Currently, ssurveillance seems to be the most important preventive strategy given the implementation in Korea and Japan of early tumor categories through nationwide screening programs with more favourable prognoses and patient outcomes [8]. The absence of an aggressive screening program may be one explanation of why, in Western countries such as the USA, 75 % of patients with upper GI cancers are diagnosed with locally advanced tumor categories and with correspondingly lower survival rates [5]. It is generally accepted that infection with *H. pylori* results in chronic inflammation, gastritis, and peptic ulcer [9]. The effects of such chronic inflammation are observed in more than 60 % of gastric cancer patients [10]. It is of molecular relevance that E-Cadherin can be observed 48 h after *H. pylori* infection in small vesicles [11] and that membrane vesicles of bacteria contain lipopolysaccharides, chromosomal deoxyribonucleic acid (DNA), plasmids, and phage DNA [12].

Studies using irradiated human skin fibroblasts have revealed the autocrine function of cyclooxygenase-2 (=Prostaglandin G/H synthetase 2, =COX-2-) dependent prostaglandin (PGE2) and cytokine production in conjunction with nuclear factor kappa-light-chain-enhancer of activated B cells (NF-κβ-) dependent gene expression of cytokines such as Interleukin 1 beta (IL-1B), IL-3, IL-6, IL-8, TNF and PTGS2/COX-2 [13]. Therefore, it appears that chronic inflammation is one of *the* important sequences in accordance with a recently proposed multistep process of carcinogenesis [14, 15].

The importance of inflammation in carcinogenesis is supported by findings showing that *H. pylori* induces PTGS2/COX-2 signaling in pre-cancerous lesions and that anti-*H. plyori* treatment results in a decrease of PGE2 levels with an observed regression of gastric pre-cancerous lesions [16]. This also explains why chronic inflammation plays a pivotal role early in cancer and why, until recently, the origin of less than 15 % of all cancers were shown to be hereditary based on the somatic mutation theory that has been predominant for some 85 years [14, 15]. From genetically derived cancers (estimated to account for some 5 to 10 % of all cancers) only about 1 % represents gastric carcinomas, 3–5 % for colorectal cancers, and about 8 % for breast cancers (breast cancer 1, early onset = BRCA1 or breast cancer 2, early onset = BRCA2) [17–19].

**Fig. 1:**
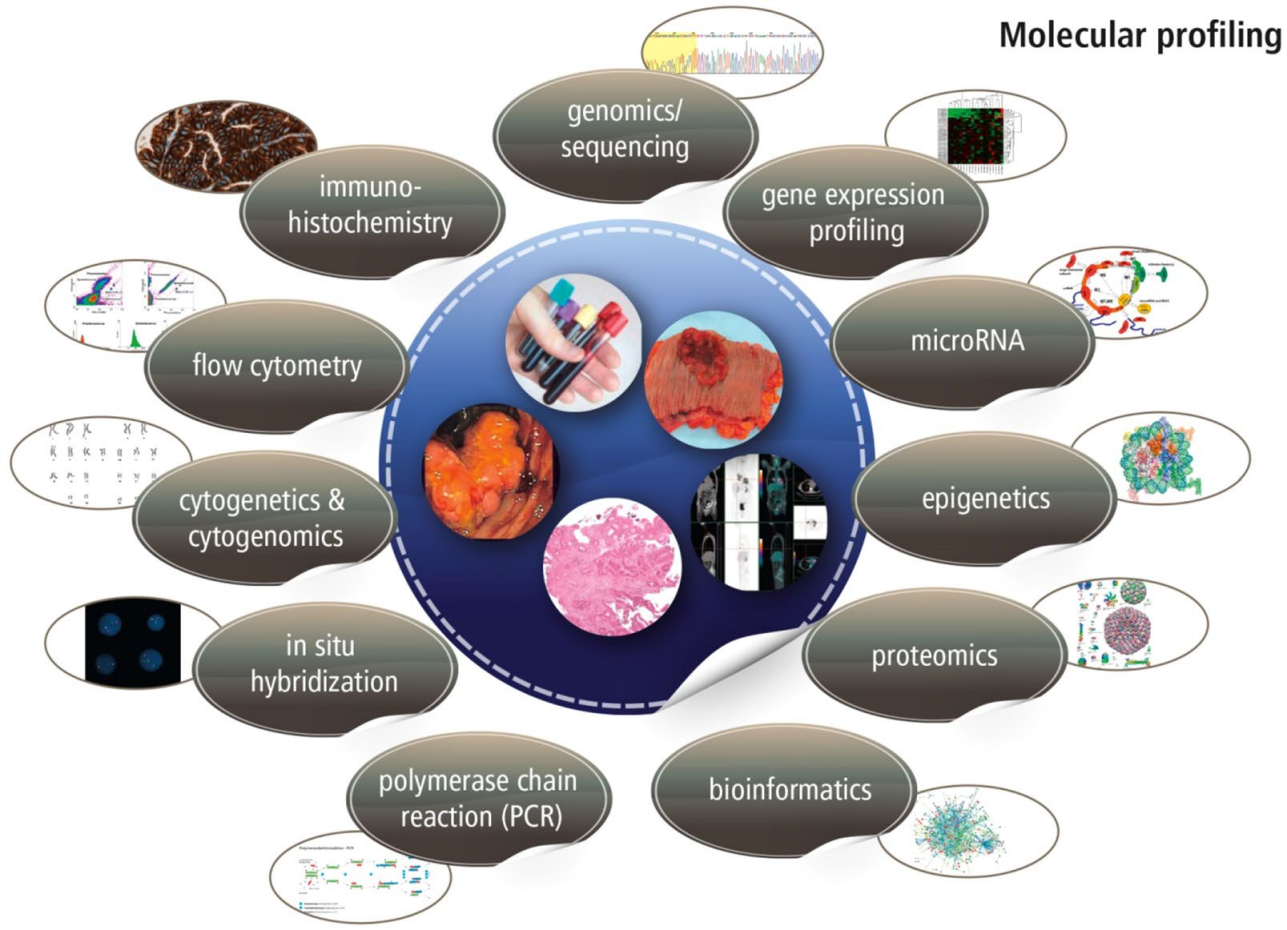
Schematic drawing of various types of diagnostic and molecular biological options for science and research (*modified according to* [22])

Recently, it has been suggested that in order to more accurately elucidate the origin of cancers, a detailed personalized and individualized conceptual model is needed in terms of both strategy and content [20]. Both seem more difficult than previously assumed as the necessity of providing available and missing evidence is required to bring about the integration of translational molecular biological data to clinical processes such as the cancer classifications proposed by the American Joint Cancer Committee (AJCC). The speed of progress in molecular biology makes such an endeavor difficult and also new technologies e.g. complex nanoparticles might will have significant influence [21]. One aspect involves evaluating available knowledge in a critical manner. Herein, we review translational data in genomics, microRNA, epigenetics and proteomics (Fig. 1) (*modified according to* [22]) for monitoring responses and, where available, patient outcomes with an emphasis on GI carcinomas.

## Review

### Genomics

#### Quantitative RT-PCR, qPCR

##### General

The invention of the polymerase chain reaction (PCR) in 1955 by Kjell Kleppe and Ian J. Molineux, and its subsequent modification by Kary B. Mullis in 1983 helped realize its potential and in so doing revolutionized biology and medicine [23–25]. PCR allows the amplification of genetic information from a few copies or a single piece of DNA. Various modifications in enzymes, quenchers, primers and protocols are used depending on the specific goal of the researcher and the questions to be addressed. However, the wide spectrum of modifications carries with it the burden of “open-to-interpretation” results and, consequently, their significance or lack thereof. Quantitative real-time reverse-transcription polymerase chain reaction (qRT-PCR) has been widely used to identify gene expression profiles in various cancers and probe differences between the genetic makeup of cancer versus normal tissues [26]. Even though qRT-PCR is perceived as less labor-intensive and a more high-throughput method than conventional RT-PCR (which involves a cDNA synthesis step) two opportunities for variability include template preparation and the dispensing of reagents. Sample collection is a crucial step in the evaluation of the gene expression profiles between tissues.

Up until recently, tissue RNA extraction was carried out from the entire gross resection and/or biopsy. Typically, these tissues consist of a large number of cells with only a small percentage being cells of interest. This is particularly important if qRT-PCR is used to determine small metastases that consist of a small number of cells (10–100 cells/site) and consequently stay undetected because of being masked by the expression level of surrounding cells. This example reveals that tissue sampling is a crucial step in the quality of data obtained and in their subsequent interpretation. Tissue sampling is performed with needles that collect a predetermined volume of the tissue of interest. This method of sample collection became more popular with the utilization of microdissection of the samples by robots that allowed for the collection of small amounts of material (as little as a single cell). However, this increase in dissection capability was not accompanied by a corresponding increase in the capability to perform qRT-PCR. Recently, a single cell RNA expression kit became available [27] but its use in high-output clinical facilities remains to be evaluated. Thus, caution has to be exercised when interpreting qRT-PCR results.

Results from this approach reflect “one” brief moment and only represent the steady state mRNA levels during the time the tissue sample was taken. Therefore, quantification of mRNA levels yields information that is measured during that single time point. No information about mRNA integrity and/or protein level is obtained.

The recently described detection of micro RNA (=miRNA, miR) introduced yet another complexity to the value of qRT-PCR namely post-transcriptional modification of translation in which mRNA level is stable with attenuation of protein synthesis. In addition, there is an influence by mRNA silencing and mRNA splitting such that qRT-PCR no longer represents being a measure of functional expression. RT-PCR data provide less usable information about protein activity or about possible mutations that the target gene might harbor unless it is in the primer binding region. Primer design can partially compensate for the problem of mutations by specifically designing primers to target small point mutations and large deletions but that requires knowing the sequences of these mutations. Therefore, for complete and biologically relevant analysis of gene expression it is necessary to complement qRT-PCR information with data that derived from immunohistochemistry and biochemical assays. Additionally, the qRT-PCR method itself is fraught with issues such as loading of reagents, variability between technicians, normalization between samples and choice of housekeeping genes. This has been reviewed in detail [28, 29].

##### Tumor heterogeneity and mutations

Intra-patient heterogeneity is a challenge for developing effective therapies. With progress in the human genome project, initial promises such as base pair resolution, genome wide and exon sequencing have become routine. Nowadays, the use of sequencing allows for the collection of enormous amounts of data with the ability to decipher the meaning of the data and attempt to translate these into the treatment of cancer patients. However, 99.9 % of all mutations that occur within the coding regions of the genome are not fully understood nor have they been thoroughly investigated. Additionally, the number of mutated genes and mutations per gene or per cancer in the coding region varies greatly [30]: Some 95–97 % of mutations are single-base substitutions and 3–5 % constitutes insertions and deletions. Furthermore, of the reported single-base mutations, some 90.7 % are missense changes, 7.6 % nonsense, and 1.7 % involves splice sites that are in non-translated regions right after the start or stop codons.

The number of mutated genes varies with smaller number of somatic mutations observed in the younger patient population in comparison with older patients with the same cancer. The number of observed mutations also varies between tissues hosting the primary cancer. Tissues with high rates of cell division, such as the colon or skin, have larger numbers of mutations per cell compared to cancers with slowly dividing tissues i.e., brain [30, 31]. The enormous variability of the mutations combined with the fact that more than 50 % of mutations occur in the cell *before* the cancer phenotype is established introduces a high *noise to signal* into genome and/or exon sequencing that confounds the interpretation of data [30, 31]. It has been assumed that mutations occur over long periods of time, in some cases over several decades and this fact alone suggests that sequencing results may vary greatly as a function of the time of sample collection.

Furthermore, it was assumed that mutations occurred after a long latency period of several decades. It has been inferred that this is a main reason for the variability in results. Recently it was demonstrated, using tumor exome sequencing, that acute lymphoblastic B-cell leukemia (B-cell ALL) is not caused by mutations but rather by infection and that mutations of Janus-activated kinase 3 (JAK-3) occur *after* carcinogenesis is already underway [32]. Thus, somatic mutations are increasingly seen as epiphenomena and subsequent events [14, 15].

An apple found in a car is not synonymous with the proof that apples grow in cars.

*The observation of a mutation within a tissue or tumor (i.e., a somatic mutation) is not synonymous with proof that mutations are causally related to the cancer.*

In contrast, a recent report suggested that oncogenes, in particular, Kirsten rat sarcoma viral oncogene (KRAS), is causally linked to de novo tumor development in human mammary cells, in vitro and when implanted in immune-deficient mice [33]. Normal human mammary cell types obtained from 37 normal human reduction mammoplasty samples included basal cells (BCs), luminal progenitors (LPs), luminal cells (LCs) and stromal cells (SCs). Such cells were exposed to encoding lentiviral preparations (encoding complementary DNAs) for TP53 and TP53-GFP (green fluorescent protein), phosphatidylinositol-45-bisphosphate 3-kinase catalytic subunit alpha (PIK3CA) and PIK3CA-YFP (yellow fluorescent protein), KRAS and mCherry-KRAS, and in some experiments, to a library of biologically neutral, barcoded lentiviral GFP vectors to allow subsequent clonal tracking of their progeny using a DNA sequencing approach. Afterwards these cells were embedded in a collagen gel and the gels transplanted into highly immunodeficient NOD-SCID or NRG female mice. The results showed that BCs and/or LPs isolated from 17 of 27 normal donors and exposed to all three oncogenic vectors produced tumors resembling invasive ductal carcinomas within 8 weeks at similar overall frequencies (46 % of BC isolates and 61 % of LP isolates). However, some major questions were not addressed such as,

- What happened in the other 54 % of BC isolates and 39 % of LP isolates?
- Why did identical treatment of LCs and SCs isolated from three of these samples not produce any tumors in the same 8-week period?

Tumors were obtained only when the KRAS oncogene was included and even on its own (64/102 = 63 % for all transductions that included KRAS) compared with 1/12 = 8 % when KRAS was not present—does this allow for the inference that KRAS is the causal factor in the observed tumors in mice? The authors suggest that their studies provide new insights into the earliest phases of malignant transformation in vivo of cells isolated directly from normal human mammary tissue. Five aspects of this study are noteworthy:

1. Rapidity and efficiency (though with high variability) with which this process can be induced using a single transducing oncogene (KRAS).
2. Considerably heterogeneity displayed in the numbers, phenotypes and growth behavior of clonally tracked human cells with tumorigenic activity in vivo within 2–8 weeks.
3. Lack of strong influence of human mammary cell type initially transduced with frequency of clones generated, the histopathology of the tumors produced or their loss of lineage-specific expression profiles. This suggests a greater effect of the potent transforming role of the KRAS oncogene in these cells. What prevents KRAS from being 100 % effective in this mouse model?
4. Frequent delayed activation of clonal growth observed in secondary tumors. This latency could either be biologically determined, reflecting an origin of these late-appearing clones from their normal counterparts with similar features, or simply reflective of a stochastic process, as previously indicated for established human breast cancer cell lines passaged in vivo.
5. If mainly basal cells, which have special location in vivo, produce cancers as proposed, why are the majority of breast cancers found in the epithelial and not in basal cells?

The research by this group provides insights to our understanding of the role of genetics in the origin of cancers, but an old dogma—the somatic mutation theory—is used to explain the results as “important” even though the clinical data tell a different story.

A recent report sheds new light on how enzymes may continuously promote mutations in cancer after the carcinogenesis process has been initiated. High levels of the DNA cytosine deaminase APOBEC3B (A3B) found in breast cancers are associated with poor survival and increased rates of resistance to tamoxifen [34]: A3B changes the microenvironment with observed *secondary* increases in mutation rates of cytosine within the estrogen receptor positive breast cell line, MCF-7L. Suppression of A3B in a xenograft model was associated with increased responsiveness to tamoxifen. It was suggested that an ongoing stimulus such as a virus, for instance, may affect an increase of A3B and that this would explain why increased mutation rates are found in locally advanced breast cancer.

Furthermore, it has been shown that a liver cancer sample of 2.5 cm (1 inch) contains more than 100 million mutations [35]. The authors showed that this high degree of genetic diversity was independent of whether the tumor sample was a thin or thick one. This observation raises the following questions:

- Do we know what mutations result in a given percentage of harm to an organism?
- If a mutation is observed, do we know *when* it occurred?
- Does the detection of mutation reveal when it was modified and/or repaired, albeit incorrectly or incompletely?
- What is the fate of any given mutation in a living organism and how do we know this?

There are other significant biological challenges as well as it has been shown that an identical mutation can result in different phenotypes [36]. We now also know that there is routine processing of mutations within the physiological context of normal growth as an integral part of development and evolution [37]. This raises the issue of how we can assume that any mutations being measured in tissue is necessarily pro-carcinogenic.

Investigating some 17 million single nucleotide variants from genomes of 562 tumors, it was shown that differential DNA repair, and *not mutations,* is the primary cause of large-scale regional mutation rate variations across the human genome [38].

By examining some 450 somatic mutations accumulated in non-repetitive genome sequences from the blood of a healthy 115-year old woman, the mass of mutations observed were harmless mutations suggesting that “*the finite lifespan of* hematopoetic stem cells *(HSCs), rather than somatic mutation effects, may lead to hematopoietic clonal evolution at extreme ages*” [39]. Another report analyzed 4742 tumor-normal pairs across 21 cancer types; the data set consisted out of “*3,078,483 somatic single nucleotide variations (SSNVs), 77,270 small insertions and deletions (SINDELs) and 29,837 somatic di*-*, tri*- *or oligonucleotide variations (DNVs, TNVs and ONVs, respectively), with an average of 672 per tumour*–*normal pair. The mutations included 540,831 missense, 207,144 synonymous, 46,264 nonsense, 33,637 splice*-*site, and 2,294,935 non*-*coding mutations*” [40]. These authors found *145,000 genetic variations per cancer type*. Thus, it would appear that somatic mutations are likely an epiphenomena and/or constitute events that occur after carcinogenesis has begun [14, 15, 41, 42], as there are also cancers which are not associated with mutations [43, 44].

The importance of these somatic mutation data is mechanistic in that these serve to trace metastasis and to evaluate entire pathways since proteins with translated mutations interact with the other proteins that may or may not be mutated. In this regard, the influence of activated signaling pathways in the cytoskeleton seems to be particularly relevant. The metastasis of epithelial tumor cells is exemplified by Syndecan-4 as the actin cytoskeleton and cell contractility are modified by a second signal path (PKC) [45].

Another variable which influences our understanding is that proteins with translated mutations can result in interactions with other proteins. For example, point mutations occur at a similar rate in cancer and non-cancerous cells and larger genetic material rearrangements such as translocations and changes in chromosome numbers, occur more frequently in cancer cells than in noncancerous cells which support the importance of the timing of sampling [46]. Despite these important challenges of intratumoral, intermetastatic, intrametastatic and inter-patient variability, some important findings have been obtained by sequencing methods, primarily that more than 138 driver mutations identified to date can be divided into pathways involved in cell survival, cell fate, and genome maintenance [30].

##### Sequencing

Transcriptome sequencing analysis, also known as RNA-seq, has been used in cancer research for the detection of transcribed mutations and confirmation of known and unknown mutations. RNA-seq is performed on the isolated total RNA from a tumor versus control samples in order to determine the differences between the two [47]. Research in prostate cancer has revealed seven new cancer-specific gene fusions, two involving the *E26 transformation-specific* or *E-twenty-six transcription factor family* (ETS) genes, *ETS translocation variant 1* (ETV1) and *ETS* related gene (ERG), and four involving non-ETS genes such as *CDKN1A* (p21), cell surface glycoprotein encoded by *CD9* gene (CD9), and *IKBKB* (IκK-beta) [48]. Using two different technologies for confirmation provides important fusion proteins like LnCap and VCaP in prostate cancer cell lines followed by identification of those proteins in the patient samples [49]. The downregulation of protein tyrosine kinase 6 (PTK6) in esophageal squamous cell carcinoma (ESCC) has been described [50].

Combining these data using bioinformatics might provide a better understanding of mutations and their role in cancer progression and might also provide insight into how miRNA functions as post-transcriptional regulators of gene expression in cancer progression [51].

## MicroRNA

MicroRNA (miR, miRNA) are small (20–24 nucleotides long) well-conserved RNA molecules involved in the control of translation of mRNA in the cell. miRNAs associated with cancers are called oncomirs. Their discovery occurred in 1993 by Ambros, Lee and Feinbaum in the nematode, *Caenorhabditis elegans* [52]. Since then, the role of miRNAs has been described in several human cancers [53]. There are more than 800 identified miRNAs [54] and their expression patterns vary in different cancers [55]. Altered miRNA expression has been reported for hepatocellular carcinoma [56, 57], pancreatic cancer [58, 59], breast cancer [60, 61] papillary thyroid cancer [62], chronic lymphocytic leukemia [63] and esophageal cancer [64–66]. The results, however, showed that up- and/or down-regulation of the miRNA of interest is usually small when comparing cancerous vs. noncancerous tissues [64, 67]. miRNAs can play different roles in esophageal cancer tumorigenesis exhibiting both pro- and anti- proliferative roles and are differentially expressed in squamous cell carcinoma and in adenocarcinoma, with and without Barrett’s metaplasia [68].

miRNAs have been detected in tissues using qRT-PCR and in situ hybridization [69]. In frozen tissue samples, 509 mature miRNA assay identified several miRNAs distinctively expressed in tumor cells when compared to corresponding normal tissue, hsa-miR-103/107 complex, in particular, showed a strong correlation between low expression and high survival periods [70]. miRNA-21 has been proven to control proliferation in vitro and in humans [69, 71]. Although it was suggested, that miRNA is part of the cancer secretome, miRNA is not a protein and therefore cannot be a part of any protein group. These molecules end up in serum and in exosomes. The term *secretome* was coined by Tjalsma in 2000 defining the secreted proteins in *Bacterium subtilis* [72] and, in 2010, Agrawal suggested using this term for ‘the global group of proteins secreted into the extracellular space by a cell, tissue, organ, or organism [73].

In the serum of patients with squamous cell carcinoma vs. patients with benign disease without inflammation, researchers detected exosomes that contain miRNA-21 [74, 75]. Serum samples depleted of exosomes did not have PCR detectable levels of this miRNA. MicroRNA-21 has been detected in the serum of patients with ESCC (100 % in one series) and its level in serum was shown to be dependent on the presence of the tumor. After resection, serum levels of microRNA-21 dropped [74, 75]. More clinical trials with larger patient populations are needed in order to fully explore the significance of microRNA-21 as a marker and predictor of responses to treatment. So far it is not known if free or exosomal miRNA has the most value as a biomarker. However, recently the potential to discriminate between precursor metaplastic Barrett’s mucosa from adenocarcinoma using miRNA was reported [76].

Transfection of colon cancer cell lines with miRNA-21 led to increases in downregulation of programmed cell death protein 4 (PDCD4), transforming growth factor beta receptor 2 levels of beta-catenin, TCF/LEF activity, and expressions of c-Myc, Cyclin-D, which are increased in cancer stem cells (CSCs) and where these are accompanied by an increased sphere-forming ability in vitro and tumor formation in SCID mice [77]. In liver regeneration, it is shown that miRNA-21 regulates rapid translation of G1/S-specific Cyclin D1 (Cyclin D1 and, consequently, increases cell proliferation [78]. MicroRNA-22 is also interesting from a response-to-therapy perspective. Researchers did not find a correlation between levels of miRNA-22 and overall survival but did find a correlation between its levels in tissue and stage of the tumor as well as in the tumor’s response to radiation therapy. Cancers with higher expression of this particular miRNA responded better to radiation therapy than cancers with lower expression of miRNA-22 [79].

Despite the promising aspects of miRNAs in cancer diagnosis and treatment, there are several obstacles for successful implementation of bench findings into the clinic. One such obstacle is detection of miRNA. Initially, Northern blotting techniques were used for miRNA discovery and detection. This method is relatively insensitive to changes in expression of miRNA and requires large amounts of starting material (RNA), usually obtained from tumor resections. Quantification of miRNA is therefore routinely analyzed by RT-PCR methods such as the modified Invader assay [80] and confocal laser-induces fluorescence detection [81], oligo-array based techniques [82] and in situ hybridization [83, 84]. It is of note that none of these techniques have been adequately validated and that their use in laboratory settings is questionable. For example, RT-PCR is semi-quantitative and can provide information on differences between cancers and noncancerous tissues; however, this technique has drawbacks as discussed below in greater detail. Although, miRNA microassays are suitable for large-scale screening, they are semi-quantitative and lack sensitivity to discriminate between small differences. In addition, adjustments of the conditions of the assay have to be balanced for large number of miRNAs which adds to variability in the efficacy of the method. Until now, only certain miRNA species have been preliminarily demonstrated as biomarkers in a clinical setting [85–87].

There are several questions to be answered before miRNAs can be widely used in the clinic. First, we have to fully understand the role of miRNAs and their biological effects. Usually, miRNA binds to multiple mRNA targets [88, 89] and, by such binding, can “label” mRNA to be degraded or translated into protein in an attenuated fashion making it extremely difficult to evaluate the importance of charges in expression [88, 90]. There are efforts underway, using bioinformatics, to model cellular responses to changes in level of particular miRNAs but with no definite conclusions as of this writing. The situation is even more complicated when hyper/hypo-methylation, histone/DNA interactions are included as we have even less mechanistic information to be able to evaluate the resulting data.

For example, in breast cancer cell lines, after 5 h of exposure to a pro-apoptotic dose of LAQ824, a small molecule histone deacetylase inhibitor, changes were measured in 40 % of the >60 different miRNA species expressed in SKBr3 cells with 22 miRNA species shown to be down-regulated and five miRNAs up-regulated [91]. This is a much higher percentage than the 5 % of affected miRNA. What is the significance of this discrepancy between the levels of miRNA and mRNA? How they interact with each other is yet to be understood. Rather than a target for intervention, certain miRNA species may serve as a diagnostic or as a companion diagnostic indicator.

### Polymorphisms

The investigation of genetic polymorphisms is important as two or more different phenotypes may exist within the same individual. Biologists investigate certain point mutations in the genotype such as single-nucleotide polymorphisms (SNPs) or variations in homologous DNA by restriction fragment length polymorphisms (RFLPs). Such investigations are performed by chromatography, chromosome cytology, or genetic data. The mechanisms and the distribution of different polymorphisms in different genes are not well understood although these are believed to be a reason for evolutionary disparity for natural selection [92].

Independent from its role in understanding biology and especially tumor biology, the investigation of polymorphisms should not be expected to reveal clinically meaningful data anytime soon. The reasons for this may be understood by considering the following information:

1. We do not understand how polymorphisms reflect a disease and/or respond to a treatment, or if they react in conjunction with polymorphisms of other genes.
2. The number of SNPs, published on 23 July 2013 in the single nucleotide polymorphism database (dbSNP), was 62,676,337 [93].
3. Polymorphisms need to include data on the generation time which is approximately 3 years; the human genome consists of base pairs (6.4 billion, 6.4 × 10^9^ base pairs) and it was assumed, that some 192 mutations (6.4 × 30) per cell generation occur. For example, within the Y chromosome this rate was estimated to be between 100 and 200 [94].
4. Humans have 23 paired chromosomes (46 chromosomes) and the human genome project revealed that humans probably have 21,000 haploid coding genes with approximately 3.3 × 10^9^ base pairs [95].
5. Chromosome 1 alone with its 249,250,621 base pairs has some 4,401,091 variations [96].
6. Mutations occur at an estimated of around 10^−6^–10^−10^ in eukaryotes [97]; this allows for an approximation for calculating the possible options and/or combinations.
7. The number of pseudogenes is about 13,000 [95].
8. A wide variation is reported in transposable (mobile) genetic DNA sequences [98, 99]. For example, Alu has about 50,000 active copies while LINE-1 (long interspersed element 1) has approximately 100 active copies per genome.
9. To the best of our knowledge, mobile genetic elements, CLASS I DNA transposons as LTRs (long terminal transposanable retroposons) and non-LTR retrotransposons, as long interspersed elements (LINE), short interspersed elements (SINEs) and CLASS II DNA transposons account for more than 40 % of the total genome [100].
10. We have additional genomic material such as the mitochondrial genome, with little understanding of how these interact with genetic elements in the nucleus.
11. The mitochondrial genome is separated from the nuclear DNA by the nuclear double membrane; however, intranuclear genomic rearrangement takes place frequently by transmission of mitochondrial DNA into the nuclear genome and especially when a normal cell has undergone transformation into a cancer cell [101].
12. Additionally, we have coding and non-coding DNA (98 % of human genome) as well as pseudogenes [95] but are not aware how these interact in any meaningful detail.

Therefore, logically and computationally it seems unlikely to think that a needle in this huge haystack might be found in the near future that could help in treating cancers but there is hope that Big Data might make a difference when the requirements for Big Data projects are addressed [102].

## Epigenetics

The main obstacle to sequencing is epigenetic changes which appear to be relevant for understanding cancer occurrence and progression. Epigenetic alterations are, by definition, mitotically and meiotically heritable changes in gene expression that are not caused by changes in the primary DNA sequence. The epigenetic modifications described in the literature generally comprise histone variants, post-translational modifications of amino acids of histone proteins, and changes in the methylation status of cytosine bases (C) in the context of CpG dinucleotides within the DNA itself. Methylation of clusters of CpG dinucleotides (CpGs–called “CpG-islands”) in the promoter region of genes have been associated with heritable gene silencing [103]. Detailed information about modified residues of one or two histones are available at UniProt [104].

A role for epigenetic factors has been shown for several cancers including esophageal cancer [105–107]. Methylation and de-methylation processes are dynamic, and efforts to correlate “methylation fingerprint” with stage of the disease suggest that there are variations in methylation that could be potentially relevant and which correlate to the stage of the disease. Each stage undergoes unique epigenetic changes at different steps of disease progression in esophageal adenocarcinoma, suggesting a step-wise loss of multiple protective barriers against CpG island hypermethylation.

Hyper- and hypo-methylation have distinct roles in the cell making the fingerprinting of cancer cells complex. Hypomethylation usually introduces genome instability and genetic rearrangements while hypermethylation silences various tumor suppressor genes [108]. The aberrant hypomethylation occurs at many different loci suggestive of an overall deregulation of methylation control in tumorigenesis in esophageal adenocarcinoma. However, there is no evidence for correlation of a distinct group of tumors with a CpG island methylator phenotype [109]. Efforts were made to investigate methylation patterns of DNA in the plasma of cancer patients vs. controls such as p16 promoter methylation in ESCC [110]. For example, the beta-catenin signaling pathway Wnt modulator *secreted frizzled-related protein 1* (SFRP1) gene is silenced by hypermethylation in ESSC [111] and the COX-2 promoter region is silenced in some ESSC cell lines [112].

The Ras-related protein Rab25 gene implicated in endocytic recycling of integrins and suppressor of invasion and angiogenesis was significantly downregulated in ESCC tumor specimens. Rab25 correlated with decreased overall survival and was also documented in ESCC cell lines as compared to pooled normal tissues [113]. Demethylation treatment and bisulfite genomic sequencing analyses revealed that downregulation of Rab25 expression in both ESCC cell lines and clinical samples was associated with promoter hypermethylation [113]. Further characterization of Rab25 may allow its use as a prognostic biomarker for ESCC and a plausible target in ESCC treatment.

Despite encouraging data, caution must be taken interpreting these results. For example, these studies typically utilized PCRs specifically designed to detect hyper- or hypo- methylation of the DNA with all the downfalls of this method. Also, the origin of the DNA detected by this method is uncertain. There is no evidence whether this DNA originates from dying “normal” cells or from cancer cells or from both.

Alterations in levels of cell-free DNA in plasma or serum as well as increases in the overall level of cell-free DNA is not restricted to any particular tumor site, type or grade. However, there are larger amounts of cell-free DNA in patients with late stage disease and metastasis. Some studies show a correlation between resection of the cancer with diminished levels of cell-free DNA in sera [114]. However, these studies have been conducted on small patient populations and require further investigation to validate their utility [115].

## Proteomics

The measurement of proteins in cancerous vs noncancerous tissues termed “cancer profiling” is a promising area for both biomarker discovery and growth in applications for healthcare. Molecular profiling for different biomarkers in a tissue may assist in obtaining more accurate diagnosis for a cancer on a personalized basis providing better information on anti-cancer treatments since every cancer cell appears to have its own pattern of active genes and proteins [116–118]. To achieve this, it will be necessary to collect information on which altered gene expression will result in what kind of final products (proteins) such that the results can be used for diagnostic or for monitoring the efficacy of therapy.

Advances in mass spectrometry (MS) have made it possible for large-scale analysis of the entire proteome of a given tissue to enable the identification of proteins in general and specific marker proteins expressed in that tissue in particular. Multiple platforms and technologies have been utilized including MS-based and antibody-based analyses. Two-dimensional gel electrophoresis (2DE) and liquid chromatography (LC) combined with tandem mass spectrometry (LC/MS/MS) are commonly used as MS-based proteomic approaches. Microarray and immunohistochemistry (IHC) are major platforms in antibody-based analyses.

To be able to find proteins that are differentially expressed in cancerous tissue only, tissue proteomes among normal, precancerous, and cancer patients were analyzed and compared. These tissue samples were obtained via resection and/or biopsies [119–121]. Body fluids including serum and plasma might be better choices not only for discovery studies but also for evaluation of patient’s response to a therapeutic reagent since they can be accessed readily [122, 123]. On the other hand, cancer cell lines were developed and analyzed to identify proteins differentially expressed in vitro, which provides an easier and faster way to discover and develop cancer-specific biomarkers [124, 125].

With respect to the proteomic study of the upper GI tract, a 2DE database for healthy human stomach tissue was reported in 2002 [126]. The authors analyzed both entire homogenate sand soluble fractions of the stomach. Over 600 protein spots were resolved in the 2DE separations. In 2010, Paulo and coworkers identified 134 proteins from normal gastroduodenal fluid using 2DE combined with LC–MS/MS [127]. Such studies can contribute to identifying disease-specific proteins when a diseased sample of the same tissue is analyzed. Likewise, using high-throughput and large scale proteomics technologies for finding upper GI cancer-specific marker proteins have been carried out over the past decade. Hundreds of proteins have been identified from various cancer patients and cell lines [119, 128–130].

A variety of samples from esophageal cancer/esophageal squamous cell carcinoma (ESCC) patients were analyzed and compared with normal tissue. With 2DE combined with MS platform, transglutaminase 3 (TGM3), heat shock protein 70 (Hsp70), TPM4-ALK fusion oncoprotein 2, myosin light polypeptide 6, keratin I and calreticulin were identified as over-expressed in tumor tissues [120, 131]. Using antibody-based technologies, over expression of protein budding uninhibited by benzidazoles 1 homolog beta (BubR1), mitotic arrest deficient-like 1 (Mad2), NF-kappaB-activating kinase, caspase 10, activator protein-1, alpha-actinin 4 (ACTN4), 67 kDa laminin receptor (67LR), COX-2, p53, secret protein acidic and rich in cysteine (SPARC), migration-stimulating factor (MSF), and vascular endothelial growth factor C (VEGF-C) were shown to be associated with ESCC [132–135].

Using an esophageal cancer cell line, proteins over-expressed included hsp70, peroxiredoxin-5, non-muscle myosin light polypeptide 6, keratin 1, annexin A4, keratin 8, tropomyosin 3, stress-induced-phosphoprotein 1, albumin, hsp70 protein 9B precursor, solute carrier family 44 Member 3, heterogeneous nuclear ribonucleoprotein L (hnRNP L), eukaryotic translation initiation factor 4A isoform 2, triosephosphate isomerase1 (TPI), peroxiredoxin1 (PRX1), forminotransferase cyclodeaminase form (FTCD), fibrinogen gamma-A chain precursor, kinesin-like DNA binding protein, lamin A/C, cyclophilin A (CypA), and transcription factor MTSG1 [136–138].

Most of the proteins differentially expressed in upper GI cancers were identified in ESCC cases. Only two proteins, apoC-I and apoC-III, were found to be elevated in the serum of patients with stomach cancer [139]. Serum amyloid A (SAA) was found in rat plasma injected with a stomach cancer cell line SC-M1 and was shown to be up-regulated in human stomach cancer [140]. These findings reinforce the new understanding that chronic inflammation is one of the key sequences in carcinogenesis [14, 15].

Large-scale profiling of cancer targets using a proteomics approach has been recognized and this may be a tool for seeking novel markers in the future. Until recently, different groups applied very different technologies in the mapping of novel upper GI cancer-related proteins and this resulted in a challenge for interpreting the disparate results. A number of proteins were identified as over-expressed in cancers and they may be valuable for future diagnosis and prognosis. For example, ACTN4 and 67LR were found to be up-regulated from stage I to III ESCC, where ACTN4 was associated with advanced tumor stage and lymph node metastasis whereas 67LR was correlated with an advanced tumor stage [133]. It is suggested that both ACTN4 and 67LR may be useful for the classification and evaluation of progression of ESCC and serve as targets for therapy. The levels of VEGF-C expression in gastro-esophageal junction adenocarcinoma were also found to be associated with stages of the tumor and lymph node metastasis. To this end, VEGF-C may be a potential biomarker for diagnosis of lymphatic metastasis and prognosis of survival in cardia carcinoma patients [135].

MSF was identified on the surface of human esophageal cancer endothelial cells (HECECs) and its antibody showed suppression of migration and adhesion of HECECs on a fibronectin matrix first induced by MSF. Furthermore, a biodistribution assay demonstrated that this antibody specifically homed into the xenograft with humanized blood vessels and suppressed tumor growth by inhibition of tumor-related angiogenesis. This observation, if it holds up in vivo, suggests that MSF may be an anti-angiogenic target for treatment of esophageal cancer [134]. TGM3 may be also a prognostic biomarker and may provide strategies to prevent recurrence of ESCC [131]. Excision repair cross complementing group 1 (ERCC1) may also be of prognostic value in multimodal treated upper GI cancer patients [141].

Hsp70 was found to be over-expressed in both cancer tissue samples and cultured cancer cells by several different groups [120, 137, 138] making it a more robust marker. As a known chaperone, Hsp70 plays a crucial role in forming and recycling nucleocytoplasmic transport receptors via direct interaction with the nuclear pore complex. Consequently, it regulates the transport of proteins between nucleus and cytoplasm. Multiple reports revealed its over-expression in esophageal cancers [120, 137, 138]. Thus, Hsp70 appears to be involved in the progression of esophageal cancers. Its impaired expression combined with the inability to transport macromolecules between the nucleus and the cytoplasm, Hsp70 may serve as a biomarker for esophageal cancer [137].

Juan and coworkers introduced five human stomach cancer cell lines (SC-M1, HONE-1, CC-M1, OECM1, GBM 8401) into nude mice separately [140]. After incubation, plasma was collected, analyzed, and compared with plasma from control mice injected with phosphate-buffered saline. In spite of some acute phase proteins found in the plasma of all mice bearing cancer cells, SAA was found over-expressed only in mice with the stomach cancer cell line, SC-M1 [140]. The authors suggested that SAA may be a specific diagnostic marker for patients with gastric cancer.

Using any protein with elevated levels as a therapeutic target may, in most cases, be a overly optimistic approach given the lack of progress in the recent past using such approaches. So far, only Her2 inhibition in Her2-type breast cancer has a positive effect in a fair proportion but not all of these patients. Elevated levels of a protein may serve well as a biomarker but it does not automatically follow that it will also serve as an adequate therapeutic target. The scientific community might put hope over outcome in expecting plausible leads for therapeutics to emerge without an in-depth understanding of the science underpinning the field.

Besides proteins that are up-regulated in esophageal cancers, several down-regulated proteins were also identified. These included subunit alpha type-3 of proteasome, calpain small subunit 1, eIF5A-1, S100-A8 protein, annexin A1, annexin A2, regulatory subunit of dehydrogenase 1calpain, glutamate, histone deacetylase 10 isoform beta, disulfide-isomerase ER-60 precursor, beta-tropomyosin (TMbeta), myosin light chain 2 (and its isoform), myosin regulatory light chain 2, and peroxyredoxin 2 [120, 136–138].

The increase of newly discovered proteins presents both a challenge and an opportunity. In particular, the expression level of two proteins, periplakin and clusterin, were nearly zero in esophageal cancers when compared to healthy tissue [142, 143]. These findings were based on Western Blot and IHC analyses without any quantification data. We may be able to use periplakin and clusterin to assess changes in patients with esophageal cancers. However, their scientific significance can be assessed only when their values in normal tissues are defined. The levels of periplakin were found to have shifted from the cell–cell boundary of normal esophageal epithelial cells to the cytoplasm of epithelial cells in early esophageal cancer, then to have disappeared completely in advanced esophageal cancer [142]. This might be encouraging in that periplakin may not only be useful as a diagnostic biomarker but also a marker for the staging of this cancer [142].

As reviewed above, many groups have investigated different potential biomarkers in upper GI cancers for potential use in making an early diagnosis as well as in providing information about prognosis of these cancers. However, the major challenges for evaluating the clinical significance of any of new biomarkers remain and include:

1. The specificity of occurrence of these proteins,
2. The availability for a drug to get access,
3. Identifying and quantifying a defined usefulness in clinical applications, and
4. A standardization of the different multiple diagnostic tools as well as its investigated biorepository.

Another challenge is that new findings will require more than a few investigations and validation steps although several of them have already been verified by various immunoassays for their presence and sub-cellular locations in cancer tissues. These protein candidates include ACTN4, 67LR, VEGF_C, BubR1, Mad2, SAA, TGM3 and MSF in which both TGM3 and MSF were further investigated in relative functional analysis [131–135, 140]. Beyond the up-regulated proteins, some down-regulated proteins found in upper GI cancer cells or tissues such as annexin A1, keratin 8, annexin A2, and periplakin have also been validated [136, 138, 142].

Due to the availability of antibodies and their costs, not every new protein marker can be tested, validated, or categorized as its biological (tumor) relevance needs to be identified first. Another huge challenge for the possible identification of a protein as a potential biomarker via large-scale protein profiling is the single amino acid mutation, and/or small insertions and deletions in the coding gene. ELISA, western blotting and IHC potentially provide highly specific protein detection but those mutations, insertions and/or deletions are not readily detected. For this, a large number of antibodies will need to be generated, especially to each and every single possible combination of mutations and even conversion of these mutations on 2D or 3D structure and, consequently, the epitope structure. However, an antibody approach is not ideal to detect and to quantify protein modifications and mutations but there is hope that MS might elucidate how best to proceed.

Serum and plasma are under investigation as they are much easier to collect and more practical in clinical applications. The key issue in protein marker discovery in serum/plasma using large-scale and high-throughput proteomics technologies is that approximately 99 % of protein mass in this sample consists of essentially 20 proteins. Nearly all biomarker proteins are present at low levels with a ratio roughly at 1:10^7^, i.e. ng/ml of cancer-related proteins versus mg/ml of physiological levels of albumin [144, 145]. Protein separation prior to sample analysis is required and widely used but with variable levels of contamination or depletion of the protein of interest can occur; and thus, downstream analyses can be biased. As a result, new biomarker discovery in serum/plasma in large-scale proteomics profiling has been limited. Another drawback for use of serum in the search for cancer biomarkers is that even though we possess information on which protein from a tissue can enter the circulation, the half-life of the target protein may not be long enough for it to be detected in serum [146]. There is virtually no published data to suggest that the half-life time of one protein is the same in normal vs. cancer tissue.

Finding novel biomarkers would be an important step for early diagnosis, treatment and prognosis in oncology. The Human Proteome Project with large-scale protein profiling on novel biomarkers discovery and validation may accelerate advancements in human health given in 2005 that more than 3020 proteins exist in the current data base produced by a number of international laboratories [147–149]. This number increased up to 10,546 in 2014 [150] and some 17,000 in 2015 while approximately some 15 % of all proteins have weak proteomic evidence and/or are still missing [151]. The significance how proteomics may influence our understanding of carcinogenesis and its impact on making of clinical decisions will be based on more precise strategies, rather than searching for a needle in a haystack without first knowing what the needle looks like.

## Limitations and challenges of reproducibility

The results from in vitro studies in cell lines present a huge challenge in their evaluation and extrapolation to human cancers absent the data to make such a leap. Contamination and incorrect interpretations can result in snowball effects of erroneous secondary research [152]. The HeLa cell line, cultivated in 1951, from Henrietta Lacks, a young cervical cancer patient, is most commonly used [153]. Although HeLa is a robust cell line and has been used in more than 70,000 studies worldwide, it has been known for almost 50 years that approximately 20 % of HeLa cell lines are contaminated and such contamination could impact study results [154]. Thus, the top four cell line repositories in the US, Germany, and Japan should be validated in a standardized manner [155].

The TS gene expression has been under investigation in 25 different human ESCC cell lines, 13 from the Japanese Cancer Research Resources Bank and 12 from the Leibnitz Institute (DSMZ) German Collection of Microorganisms and Cell Cultures [156]. The IC50, a parameter used to determine how much of a substance is needed to inhibit a biological process by 50 %, ranged from 1 to 39.8 µmol/L. This finding by Ando et al. is important as it unequivocally shows that it is not enough to use a cell line for a study given that the range of IC50 s in 25 ESCC cell lines under investigation is quite variable. In keeping with this finding is more recent research comparing molecular details of cell lines to real tumors by genomic profiling [157]. These authors show that common cell lines used for research on ovarian cancer do often not reflect real tumor biology in humans. This means that the results found in cell lines, more often than not, are not representative of the real tumor biology seen in patients.

Furthermore, science faces another challenge which is that a lack of reproducibility of existing cancer research is recognized and frequently discussed. However, we need to remind ourselves that less than 20 % of highly ranked so-called “landmark” publications have been shown to be irreproducible although these are among the most cited references [158]. A recently launched initiative requires that authors provide their raw data for a validation check [159]. This, together with proposed guidelines, could be of helpful to be incorporated into a plan of action thus increasing the value and reliability of cancer research. There is hope overcoming the issues of contamination and poor reproducibility by an U.S. and European initiative, which is on its way replacing its cell lines with patient-derived xenografts (PDX).

## Conclusions

There is hope of integrating translational molecular data from genomics, microRNA, epigenetics, and proteomics to improve cancer diagnosis, therapy, and facilitate cancer classifications. Reviewing the available literature for relevance to cancer diagnosis or monitoring of treatment is presently difficult as clearly defined inclusion and exclusion criteria in [1] clinical trials as well as [2] in molecular research are largely absent. Different tumor entities with different tumor biologies, prognoses, and different therapeutic approaches make it difficult to make informed recommendations. As pointed out, many hitherto undervalued criteria such as epidemiology, embryology, and molecular biology will be important to consider when designing future research and clinical trials [4].

In order to achieve these goals, it is necessary to understand the key issues in different aspects of biotechnology so as to anticipate future directions of personalized and individualized diagnosis and multimodal treatment strategies. Providing an overview of translational data in cancers proved to be a challenge as different methods and techniques used to obtain molecular data are based on different tumor entities with different tumor biology and prognoses as well as vastly different therapeutic approaches. The pros and cons of the available methodologies and the potential response data in genomics, microRNA, epigenetics and proteomics as well as their limitations in gastrointestinal cancers are considered to allow for an understanding of where these technologies stand with respect to cancer diagnosis, prognosis and treatment.

A step towards a solution would be if organizations dealing with GI cancers create a framework for biological studies and for clinical trials dealing with tumor entities. This, together with integrating other variables such as providing raw data with a validation check would be helpful. The replacement of cell lines by patient-derived xenografts (PDX) for in vitro studies may significantly enhance the value and rigor of basic and translational research.

## Abbreviations

2DE: two-dimensional gel electrophoresis
67LR: 67 kDa laminin receptor
A3B: apolipoprotein B mRA-editing enzyme, catalytic polypeptide-like 3B (=APOBEC3B)
ACTN4: activator protein-1, alpha-actinin 4
AEG: adenocarcinoma of the esophago-gastral junction
AJCC: American Joint Cancer Committee
APOBEC3B: apolipoprotein B mRA-editing enzyme, catalytic polypeptide-like 3B (=A3B)
B cell ALL: acute lymphoblastic B-cell leukemia
BCs: basal cells
BRCA1: breast cancer 1, early onset
BRCA2: breast cancer 2, early onset
BubR1: benzidazoles 1 homolog beta
C: cytosine bases
CpGs: CpG dinucleotides
CpG-islands: methylation of clusters of CpGs
CD9: cell surface glycoprotein encoded by CD9 gene
COX-2: cyclooxygenase-2 (=Prostaglandin G/H synthetase 2)
CSCs: cancer stem cells
Cyclin D1: G1/S-specific Cyclin D1
CypA: cyclophilin A
dbSNP: single nucleotide polymorphism database
DNA: deoxyribonucleic acid
DNVs: somatic di-nucleotide variations
DSMZ: Leibnitz Institute DSMZ, German Collection of Microorganisms and Cell Cultures
ERCC1: excision repair cross complementing group 1
ERG: ETS related gene
ESCC: esophageal squamous cell carcinomas
ETS: E26 transformation-specific or E-twenty-six transcription factor family
ETV1: ETS translocation variant 1
FTCD: forminotransferase cyclodeaminase form (FTCD)
GI: gastrointestinal
HECECs: human esophageal cancer endothelial cells
hnRNP L: heterogeneous nuclear ribonucleoprotein L
HSCs: hematopoetic stem cells
Hsp70: heat shock protein 70
IC50: half maximal inhibitory concentration
IHC: immunohistochemistry
IKBKB: inhibitor of kappa light polypeptide gene enhancer in B-cells, kinase beta (= IκK-beta)
IκK: inhibitor of κK kinase
IL-1B: interleukin 1 beta
JAK-3: Janus-activated kinase
KRAS: Kirsten rat sarcoma viral oncogene
LAQ824: a small molecule histone deacetylase inhibitor
LC: liquid chromatography
LC/MS/MS: tandem mass spectrometry
LCs: luminal cells
LINE-1: long interspersed element 1
LnCap: cell line: epithelial prostate cancer cell line
LPs: luminal progenitors
LTRs: long terminal transposanable retroposons
LINE: long interspersed elements
Mad2: mitotic arrest deficient-like 1
miR: micro RNA (=miRNA)
miRNA: micro RNA (=miR)
MCF-7L: human breast cancer adenocarcinoma cell line
MS: mass spectrometry
MSF: migration-stimulating factor
NF-B: nuclear factor kappa-light-chain-enhancer of activated B cells
NOD-SCID: non-obese diabetic-severe combined immunodeficiency mice, immunodeficient mice with lack of mature T-cells, B-cells and natural killer (NK) cells
ONVs: somatic oligonucleotide variations
p21: protein 21, cyclin-dependent kinase inhibitor 1 or CDK-interacting protein 1
p53: tumor protein p53 (=TP53)
PCR: polymerase chain reaction
PDCD4: programmed cell death protein 4
PDX: patient-derived xenografts
PGE2: COX-2 dependent prostaglandin E2
PIK3CA: phosphatidylinositol-45-bisphosphate 3-kinase catalytic subunit alpha
PIK3CA-YFP: PIK3CA yellow fluorescent protein
PKC: protein kinase C
PRX1: peroxiredoxin1
PTK6: protein tyrosine kinase 6
qRT-PCR: quantitative real-time reverse-transcription polymerase chain reaction
Rab25: Ras related protein Rab-25
RFLPs: restriction fragment length polymorphisms
SCs: stromal cells
SEER: surveillance, epidemiology and end results database
SFRP1: Wnt modulator secreted frizzled-related protein 1
SINDELs: small insertions and deletions
SINEs: short interspersed elements
SNPs: single-nucleotide polymorphisms
SPARC: secret protein acidic and rich in cysteine
SSNVs: somatic single nucleotide variations
TCF/LEF: transcription family group binding to DNA
TGM3: transglutaminase 3
TMbeta: beta-tropomyosin
TNF: tumor necrosis factor
TNVs: somatic tri-nucleotide variations
TP53: Tumor protein p53 (= p53)
TP53-GFP: p53 green fluorescent protein
TPI: triosephosphate isomerase1
TPM4-ALK: TPM4-ALK fusion oncoprotein 2
VCaP: cell line: vertebral cancer of the prostate
VEGF-C: vascular endothelial growth factor C
Wnt: beta-catenin signaling pathway
